# Growth Mechanisms and Electronic Properties of Vertically Aligned MoS_2_

**DOI:** 10.1038/s41598-018-34222-z

**Published:** 2018-11-07

**Authors:** Chen Stern, Shmuel Grinvald, Moshe Kirshner, Ofer Sinai, Mark Oksman, Hadas Alon, Oren E. Meiron, Maya Bar-Sadan, Lothar Houben, Doron Naveh

**Affiliations:** 1 Faculty of Engineering and Institute for Nanotechnology and Advanced Materials, Bar-Ilan University, Ramat Gan, Israel; 2 Department of Chemistry and Institute for Nanotechnology and Advanced Materials, Bar-Ilan University, Ramat Gan, Israel; 3 Department of Chemistry, Ben Gurion University, Beer-Sheva, Israel; 4 Department of Chemical Research Support, Weizmann Institute of Science, Rehovot, Israel

## Abstract

Thin films of layered semiconductors emerge as highly promising materials for energy harvesting and storage, optoelectronics and catalysis. Their natural propensity to grow as oriented crystals and films is one of their distinct properties under recent focal interest. Specifically, the reaction of transition metal films with chalcogen vapor can result in films of vertically aligned (VA) layers, while metal-oxides react with chalcogens in vapor phase to produce horizontally aligned crystals and films. The growth mechanisms of vertically oriented films are not yet fully understood, as well as their dependence on the initial metal film thickness and growth conditions. Moreover, the resulting electronic properties and the role of defects and disorder had not yet been studied, despite their critical influence on catalytic and device performance. In this work, we study the details of oriented growth of MoS_2_ with complementary theoretical and experimental approaches. We present a general theoretical model of diffusion-reaction growth that can be applied to a large variety of layered materials synthesized by solid-vapor reaction. Moreover, we inspect the relation of electronic properties to the structure of vertically aligned MoS_2_ and shed light on the density and character of defects in this material. Our measurements on Si-MoS_2_ p-n hetero-junction devices point to the existence of polarizable defects that impact applications of vertical transition-metal dichalcogenide materials.

## Introduction

The growing interest in layered inorganic materials over the last decade was stimulated by the development of novel synthetic methods. Among such methods chemical vapor deposition (CVD) dominated the production methods of scalable, device-grade transition-metal dichalcogenides (TMDC)^1–3^. In turn, the success of the synthetic research has propelled the research on novel device applications^4–7^ and fundamental physics of layered materials^8–10^. Hitherto, CVD growth of TMDCs was mostly focused on the growth of ultrathin, few-layer crystals and heterostructures^11–15^. The recent reemergence of vertically aligned (VA) thin films of TMDCs is imperative to diverse device applications and of fundamental interest, owing to their unique growth mechanism^16–19^. Moreover, thin VA-MoS_2_ films were established as scalable, high quality electronic materials, enabling highly-responsive near infrared photodetectors^20–24^ and energy storage devices based on Li^+^ and Na^+^ intercalation^25,26^. However, the role of defects and crystalline disorder can be critically important in these materials. Solar energy harvesting with self-driven photodiodes^27^ of VA-MoS_2_ on P-Si yields relatively low quantum efficiency due to the enhanced recombination rates associated with defects in VA-MoS_2_. Catalytic activity of VA-MoS_2_ is associated with its high density of edge states and is also prone to be influenced by defects and crystalline disorder^17^. Therefore, understanding the formation and properties of defects and disorder in VA-MoS_2_ is critically important for a variety of applications and may pave the way for achieving the full potential of vertically aligned layered compounds.

Early studies of VA-MoS_2_ deposition were conducted by RF magnetron sputtering^28,29^. More recently, studies on the CVD growth mechanisms of VA-MoS_2_ found that sulfurization of thin Mo films changes character from planar to vertical crystal growth, controlled by the thickness of the Mo film^30,31^. Specifically, a critical thickness of 5 nm was found to be the threshold for vertical growth, while sulfurization of thinner metal films resulted in planar oriented MoS_2_^19^. The vertical orientation of VA-TMDCs is attributed to a diffusion-driven growth mechanism^19,32,33^. In this mechanism, sulfur diffuses through the Van der Waals interlayer gap at a much higher rate than across the crystal layers – and thus the preferred orientation of the growth is established from kinetic considerations. However, the details of the vertical alignment mechanism and the turning point from planar to vertical growth are not yet fully understood. These details are significant for the advancement of several important device applications across multiple fields^32,34–37^. Moreover, the impact of a multi-domain (vertical and randomly oriented) structure on the electronic properties of MoS_2_ remains unclear. In this work, we study the formation mechanism of VA-MoS_2_ films on P-Si and their charge carrier characteristics as resolved from transport measurements of p-n junction devices.

## Results and Discussion

**Figure 1:**
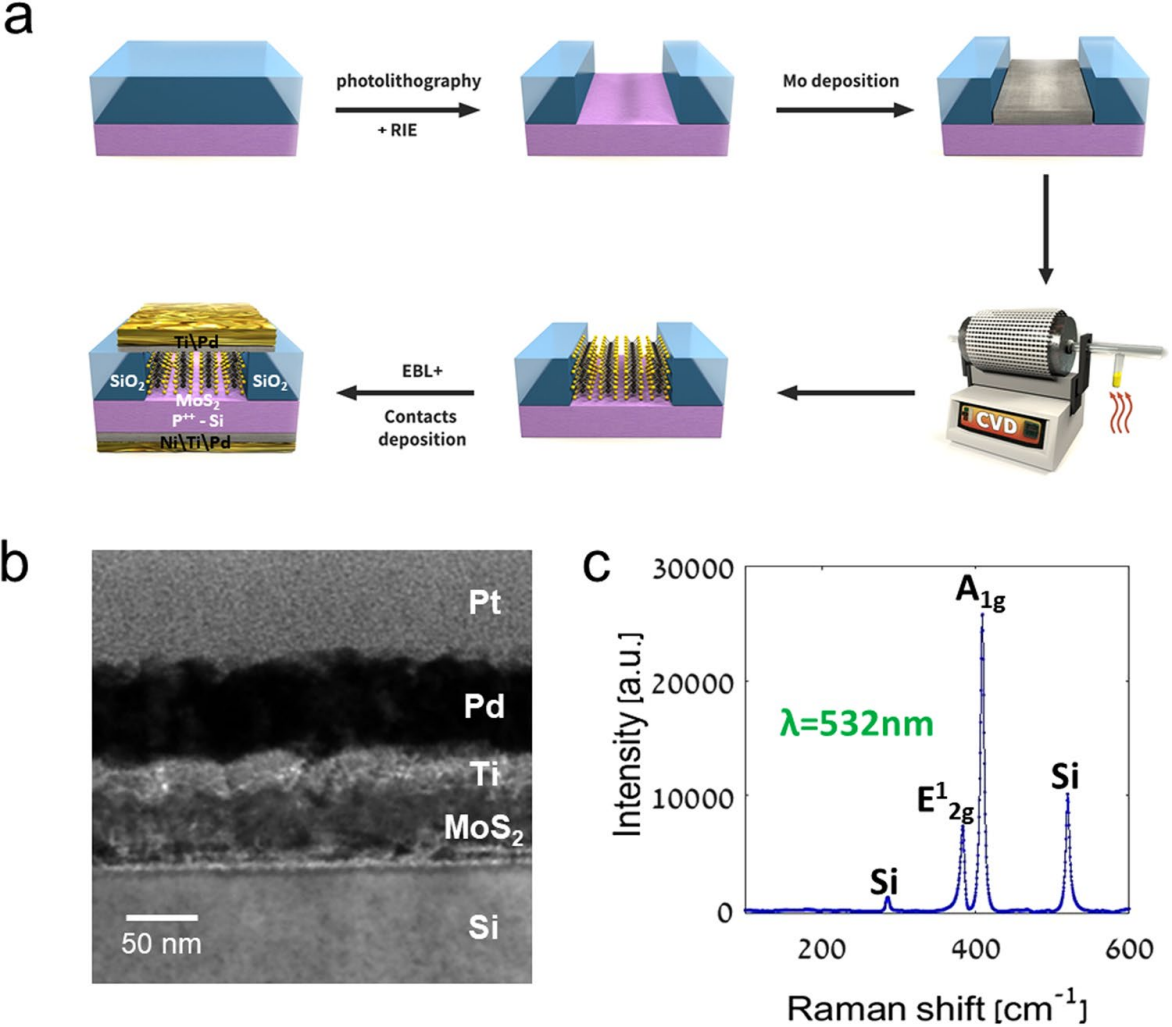
(**a**) Graphical representation of a MoS_2_ device fabrication process: 90 nm SiO_2_/Si selective etch by photolithography and reactive-ion etching, Mo deposition and lift-off, sulfurization growth of VA-MoS_2_, and metallization of devices. (**b**) A cross-sectional TEM micrograph of the resulting device. (**c**) Raman spectrum of the MoS_2_ taken immediately after growth.

Our devices are constructed from 70 nm thick MoS_2_ grown on P^+^-Si by CVD in the following process: 90 nm Si/SiO_2_ wafers were patterned by photolithography and etched to form through-oxide vias (Fig. 1a), on which 30 nm metal Mo was deposited and lifted-off. Mo films were then sulfurized under flow of 30 sccm nitrogen in background pressure of 5 mTorr, carrying sulfur vapor from a boat heated to 150 °C. The sample was kept at 800 °C during the growth. Clean Raman spectra were taken from the samples at a parallel geometry between the laser line and the c-axis of the crystal, with linearly polarized light (Fig. 1c), obtaining the signal of 2H-MoS_2_ and crystalline Si, without traces of molybdenum silicide. Then, a second lithographic process was applied for metallizing the grown MoS_2_ with Ti/Pd contacts (Fig. 1b).

**Figure 2:**
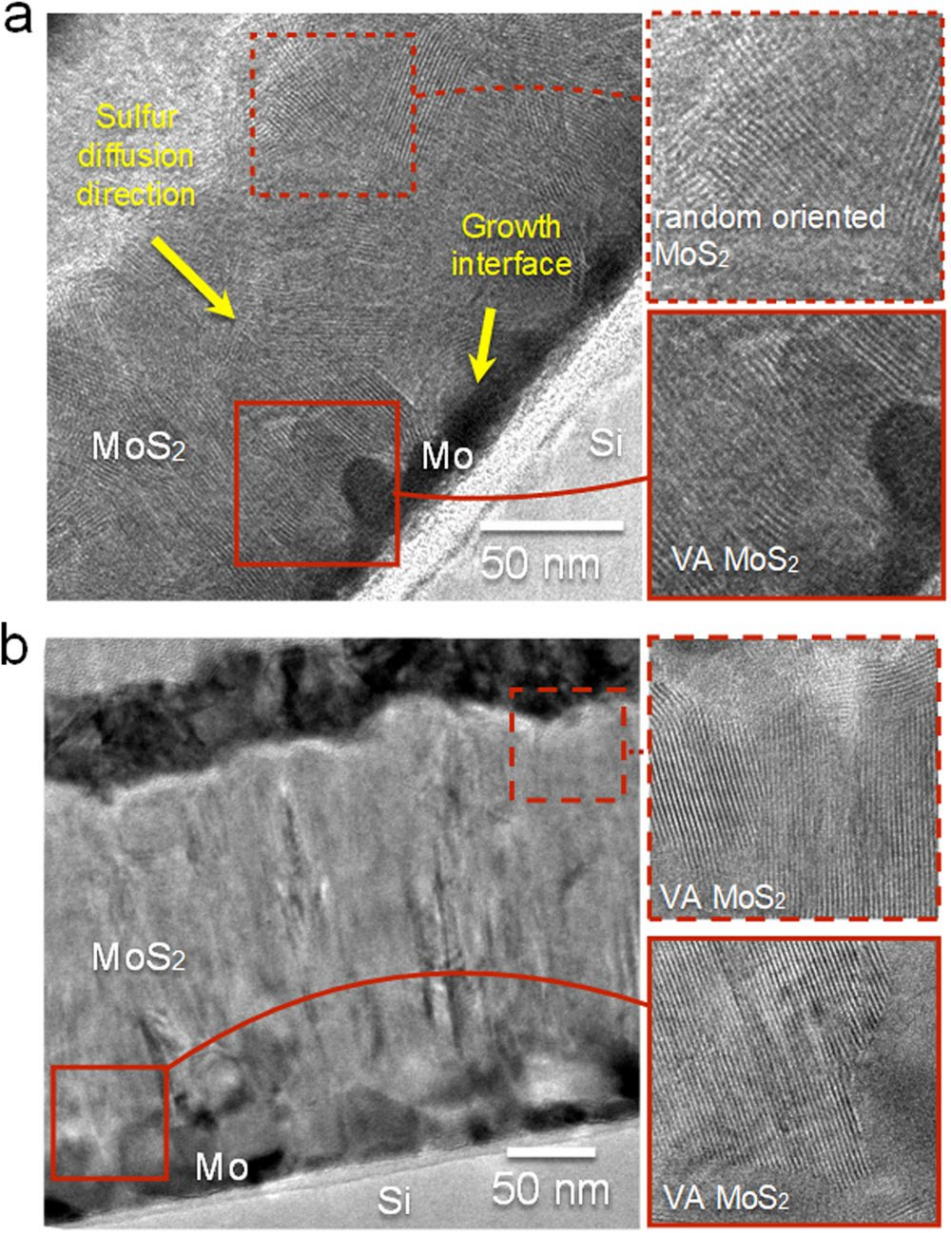
TEM micrograph of VA-MoS_2_ grown on Si substrate at 800 °C for 2 h with a temperature ramping rate of 20 °C/min (**a**) and 5 °C/min (**b**), respectively. Insets show the magnified view of vertically aligned MoS_2_ in the initial growth at the top (dashed rectangle) and at the growth interface (solid rectangle). The surface reaction results in an initial growth of a significantly thick randomly oriented MoS_2_ layer for fast growth, in contrast with slow growth where the layers are vertically aligned (dashed rectangle).

A detailed characterization of the MoS_2_ layer was performed with cross-section transmission electron microscopy of samples as shown in Fig. 2, with two types of thermal processing; in both cases a thin film of Mo on Si/SiO_2_ (as in Fig. 1) were sulfurized at 800 °C for two hours under continuous temperature ramping. Interestingly, samples of fast temperature ramping (20 °C/min) exhibit thicker domains of randomly oriented crystals as compared to slow temperature ramping samples (5 °C/min). Details of the process are given below and in the supporting information. Temperature cycles are displayed for clarity in Fig. S1. The proposed growth mechanism assumes that during the reaction, the growth front propagates into the Mo film from the vapor-solid interface inwards, finally reaching the Si substrate. Complementary, the case of thick Mo films representing the growth process at the semi-infinite samples was studied on thick Mo foils to elucidate the limit of long reaction time and depth (Figs S2 and S3), as discussed in the supplementary information. During the reaction on Mo on Si/SiO_2_, the growth front propagates into the Mo film, from the vapor-solid interface inwards, finally reaching the Si substrate. The image for the fast growth (Fig. 2a) reveals a gradual establishment of preferred crystallographic orientation. The MoS_2_ is crystalline along the reaction path, initially showing random orientation that after a threshold thickness becomes a vertically oriented layer. Thus, the crystal orientation ordering towards VA-MoS_2_ started only after an incubation thickness that we term as “surface reaction”. Following the surface reaction a strongly preferred orientation is evidenced by the sharpening of the orientation distribution around the perpendicular axis (Figs S4 and S5), in proximity to the substrate. In order to understand this growth process and the parameters controlling the thickness of the disordered layer formed at the early stages of the reaction, we quantified the crystal orientation of the films as a function of the reaction coordinate (Fig. S4). Results of the quantification procedure are displayed in Fig. S5. This transition in growth leaves a 30–40 nm thick randomly oriented layer at the fast growth conditions. In contrast, for the sample grown at slow growth conditions (Fig. 2b), the typical thickness of the surface reaction layer is much smaller than that of the fast growth (<10 nm), in agreement with previous studies^16,19,33^. Under low growth rate the disordered layer is thinner, thus indicating a lower surface reactivity and dominant diffusion-limited growth mechanism. The examples of Fig. 2 shows that the thickness of the randomly oriented film is strongly affected by the growth kinetics, yet the growth mechanisms and the role of the disordered layer were not thoroughly examined.

Here, we present a model describing the growth process resulting in the co-existence of two distinct morphologies: disordered layer of surface reaction and VA-MoS_2_. Using the model, we show the underlying principles to achieve control over the structural features and the associated electronic properties. Details of the model are described in the supporting information, and in Fig. S6. In this model the random MoS_2_ extends from the surface to a time-dependent depth *x*_0_, and the VA-MoS_2_ extends from *x*_0_ to *x*_1_. The model assumes that: (i) Mo is stationary and the reaction is driven by sulfur diffusion only; (ii) sulfur is fully reacted and no inclusions exist in the Mo film; (iii) the consumption of sulfur takes place at the moving boundary between Mo and MoS_2_, *i.e*., at the reaction front.

The set of Eq. (1) present the model for an equilibrium growth at the Mo-MoS_2_ interface^38^, while the initial and boundary conditions are fit to represent the conditions of the chemical process.

1 $$\begin{array}{rlll}\frac{\partial C}{\partial t} & = & {D}_{Mo{S}_{2}}\frac{{\partial }^{2}C}{\partial {x}^{2}},0\le x\le {x}_{0}(t) & \\  &  &  & 0 < t < \tau \\ \frac{\partial C}{\partial t}+\frac{\partial \eta }{\partial t} & = & {D}_{Mo}\frac{{\partial }^{2}C}{\partial {x}^{2}},\,{x}_{0}(t)\le x\le \infty  & \end{array}$$

**Figure 3:**
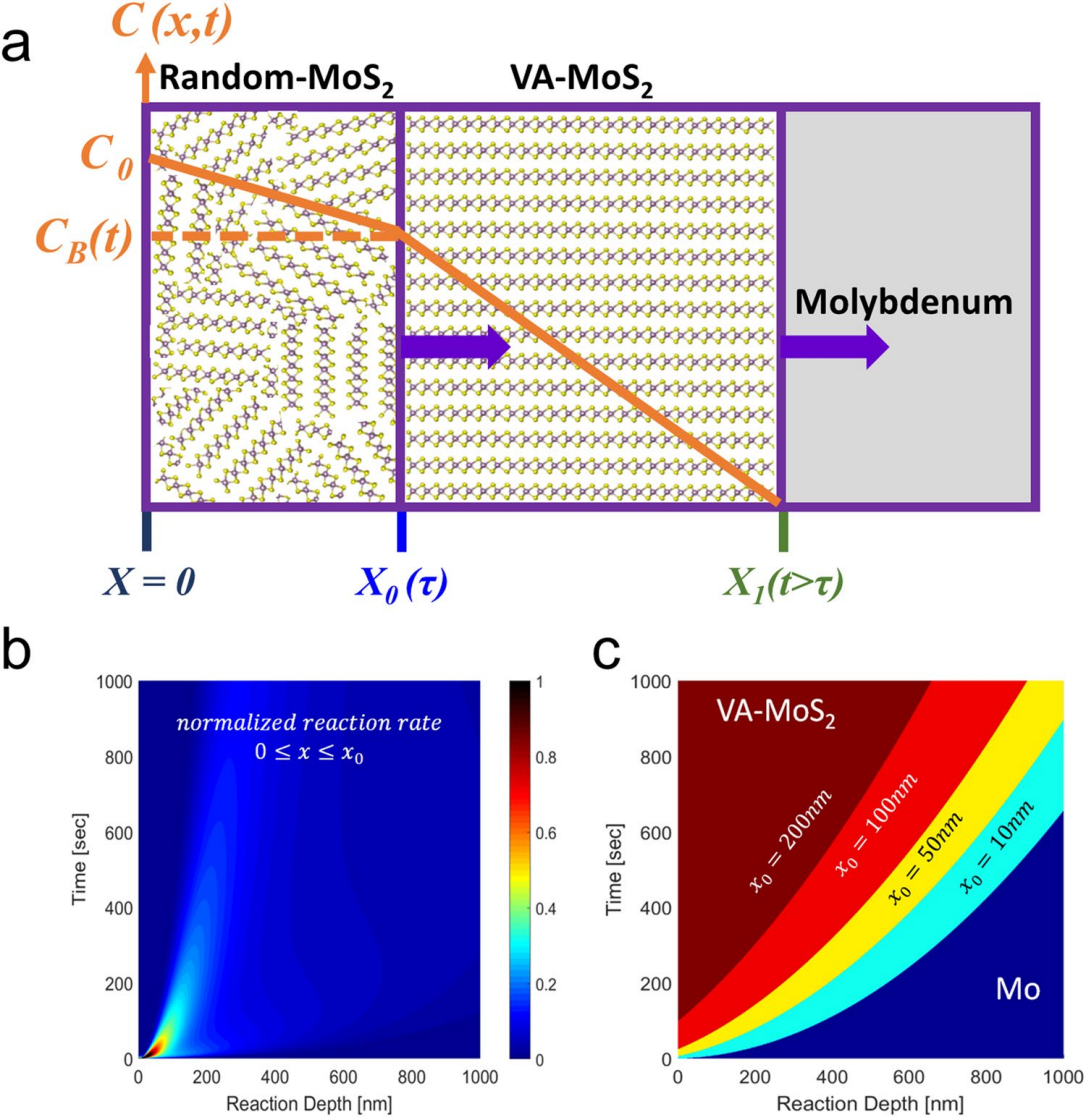
(**a**) Graphical illustration of growth process starting from a surface reaction of depth *x*_0_ and reaction time τ, followed by a diffusion-limited growth of VA-MoS_2_. (**b**) The solution to the propagation velocity of *x*_0_*(t)* with a scaled amplitude showing its rapid decay and (**c**) the resulting VA-MoS_2_ growth with several values for *x*_0_.

Here, *C* is the sulfur concentration and *D*_*Mo*_ *and* ${D}_{Mo{S}_{2}}$ are the diffusion coefficients of sulfur in Mo and in MoS_2_, respectively; *η* is the concentration of Mo consumed at the interface which is linearly related to C by the constant R by $\eta (x,t)=RC(x,t)$. *x*_0_(*t*) is the position of the reaction front, representing the moving boundary of the Mo-MoS_2_ interface. The reaction obeys the following conditions: initially, the sample comprises Mo only [*x*_0_(*t* ≤ 0) = 0]. The self-limited (see SI for details) reaction time for the growth of random MoS_2_ is *τ*: $[{x}_{0}(t\ge \tau )={x}_{0}(\tau )]$. The random layer grown within time *τ* forms a diffusion barrier and the reaction rate, *dx*_0_/*dt*- decays after *τ* (see Fig. 3b), where the reaction becomes diffusion-limited. From this point, the growth front advances through vertical seeds and is dominated by the higher diffusion kinetics of sulfur along the Van- der Waals gap that defines the fast axis within MoS_2_ with a diffusion coefficient *D*_⊥_ for sulfur. Therefore, the reaction continues along a new Mo/VA-MoS_2_ interface that we label as *x*_1_(*t* > *τ*). Figure 3a schematically displays our model at which a surface reaction advances from the molybdenum surface into its depth, up to a distance *x*_0_(*τ*)- followed by a diffusion-limited growth along the fast axis of MoS_2_ that obeys Eq. (1), with a time-dependent boundary condition *C*_*B*_(*t*) = *C*(*x*_0_, *t* > *τ*):

2 $${C}_{B}({x}_{0},\,t)={C}_{0}erfc(\frac{{x}_{0}}{2\sqrt{{D}_{\perp }t}}),t > \tau $$

Figure 3b shows the scaled reaction front velocity, *dx*_0_/*dt*, as obtained from the analytical solution (see SI for the full solution), showing that the surface reaction dominates the initial stages of the growth. Figure 3c provides the solution *x*_1_(*t*) - the thickness of VA-MoS_2_ with several values of the disoriented film thickness - *x*_0_(*τ*), demonstrating the role of this diffusion barrier layer and its impact on growth kinetics, being a barrier for sulfur diffusion. The diffusion coefficients and the parameter R can be calculated from *ab-initio* and molecular dynamics of Mo and sulfur^32,34,36,37^.

In some cases, the existence of a randomly oriented layer may be desired and can serve as a diffusion barrier in several processes, such as intercalation, that are widely spread across various applications. In case that a sharp interface is required, this layer can be removed by dry etching.

**Figure 4:**
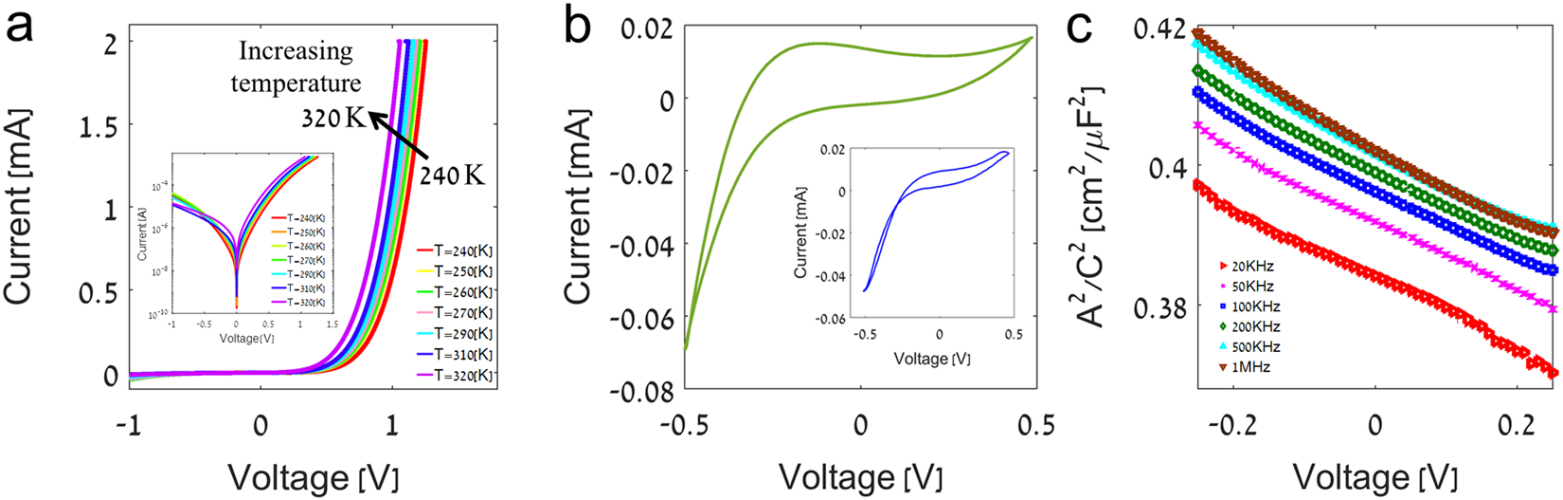
Electrical characterization of Si – VA-MoS_2_ heterostructure p-n diode. (**a**) Temperature dependent I-V curves (inset: logarithmic scale), (**b**) cyclic I-V curve at 10 Hz (inset: 100 kHz), and (**c**) voltage-capacitance curves.

It is well known that defects in MoS_2_ films are the main source for charge carriers^39–42^, which raises the question of the quality of devices made with VA-MoS_2_. The disordered layer contains unique structural features and their influence on the electronic properties is so far unexplored. To this end, we prepared diodes from VA-MoS_2_ grown on p^+^-Si (similar to the sample presented in Fig. 2a), and we confirmed that they obey the standard model of diffusive transport (Fig. 4a), *I* = *I*_0_(exp[*qV*/*ηk*_*B*_*T*] − 1). On these well-behaved diodes we could measure the electrical characteristic of the carriers and the impact of charge traps. Figure 4b shows a sizable hysteresis under cyclic bias applied on the diodes, corresponding to charge trapping at crystalline defects, rendering them polar^43–45^. By analogy with polar ions observed in perovskites^46^, we assign this electrical behavior to trap charging and discharging. The quenching of the hysteresis with increased frequency is a result of the trap capacitance and the resulting trapping/detrapping rate. From the capacitance measurements in Fig. 4c, the estimation of the built-in potential is 1.2 eV and the carrier density is ~10^19^ cm^−3^. The measured built-in potential represents the p-n quasi-Fermi level difference and the band misalignment across the junction that can be roughly estimated to ~100 meV in this case of highly doped, sharp junction. We assume that the measured carrier density is comparable with the density of traps (*N*_*t*_ ≈ *N*_*D*_) and that the vast majority of carriers in VA-MoS_2_ originate from shallow traps^47,48^.

To conclude, we analyzed the growth of MoS_2_ by sulfurization of metallic Mo films from several perspectives. We applied a high-temperature, low pressure growth on thin film Mo resulting in a gradual transition from disordered crystalline MoS_2_ to a vertically oriented morphology. By applying a theoretical diffusion-reaction model to the growth, we provided a plausible growth mechanism that explains the experimental observations. Our model is general and applicable to a large variety of layered materials. If provided with diffusion parameters retrieved from experiments or *ab-initio* calculations, it will provide a predictive, quantitative tool to characterize the growth process. Regarding the electronic properties of samples containing a disordered layer, we found that the band misalignment between Si and VA-MoS_2_ can be estimated to ~100 meV and that structural defects result in trap states that can be observed in cyclic I-V measurements. Details of the trap energy and density are a subject for future work. Yet without loss of generality, we conclude that rapid reactions result in higher defect density and that the surface reaction taking place at the beginning of the process is the source for the majority of defects. These conclusions may have critical implications to the performance of VA-MoS_2_ in several fields including catalysis, lithium ion storage and solar energy harvesting and optoelectronics devices.

## Methods

### Sample preparation

#### Si wafer preparation

Photolithography was applied to a sample of 90 nm SiO_2_/Si P-type wafer to create an array of 324 circles of 40 µm diameter each. The oxide layer in the circles was then selectively etched by reactive-ion etching, followed by evaporation and lift-off of ~30 nm Molybdenum, after which the CVD process was applied. Metal contacts were finally deposited by e-beam lithography and evaporation.

### CVD process

#### Si wafer

Samples were annealed under 160 sccm N_2_ flow at 200 °C for 1 h. Sulfur powder was placed upstream at low temperature zone of ~150 °C. The quartz furnace was heated (20 °C/min and 5 °C/min, for the fast/slow temperature ramping samples, respectively) to 800 °C under 30 sccm nitrogen flow and were held at this set-point for 2 h. Then the temperature was reduced to 600 °C, the sulfur was solidified and afterwards the temperature was reduced (7 °C/min until 400 °C and afterward 2 °C/min till reaches to room temperature).

#### Mo foil

The sample was annealed under 160 sccm N_2_ flow at 200 °C for 1 h. Sulfur powder was placed upstream at low temperature zone of ~150 °C until liquefied. The quartz furnace was heated to 500 °C (20 °C/min) under 100 sccm nitrogen flow and was held at this set point for 30 min. Subsequently the quartz furnace was heated to 600 °C (20 °C/min) under a nitrogen flow of 100 sccm and it was held at this set point for a period of 30 min. An additional heating step was added while the temperature rose to 750 °C (20 °C/min) under a 30 sccm nitrogen flow. This state was maintained for a period of 24 h. In the last thermal cycling step the temperature was reduced to 600 °C, the sulfur was solidified and the temperature was further reduced to 400 °C (5 °C/min) and then brought down to room temperature (2 °C/min). Further details and a graphical representation of the temperature cycle are presented in the supporting information.

#### TEM

TEM lamella of the CVD-grown VA-MoS_2_ devices were prepared using a FEI Helios 600 Focused Ion Beam (FIB) microscope. Pt was deposited during the FIB process for the purpose of material protection. TEM micrographs of the cross-sectional device samples were collected on a JEOL JEM-2100 high resolution transmission electron microscope operated at 200 kV.

#### Raman Spectroscopy

Raman spectra were taken by a Horiba Scientific Labram HR Evolution using a 532 nm laser. The exciting laser propagated parallel to the crystal c-axis with a linear polarization.

#### Electrical measurements

Samples were wire bonded to ceramic leadless chip carrier, and then were measured in a cryostat vacuum chamber (Montana instruments) with a Keysight B2912A and B1500A instruments.

## Electronic supplementary material

Supplementary Information
