# Supplementary material for: Growth Mechanisms and Electronic Properties of Vertically Aligned MoS2
Source: Sci Rep. 2018 Nov 7;8:16480. doi: 10.1038/s41598-018-34222-z (PMC6220198; doi:10.1038/s41598-018-34222-z)
Supplement: Supplementary file 1 — Supplementary Information [file 41598_2018_34222_MOESM1_ESM.pdf]

Supporting Information

Growth Mechanisms and Electronic Properties of Vertically Aligned MoS_2

Chen Stern,[1] Shmuel Grinvald,[1] Moshe Kirshner,[1] Ofer Sinai,[1] Mark Oksman,[1] Hadas Alon,[1,2] Oren E. Meiron,[3] Maya Bar-Sadan,[3] Lothar Houben,[4] and Doron Naveh[1,*]

[1] Faculty of Engineering and Institute for Nanotechnology and Advanced Materials, Bar-Ilan University, Ramat-Gan, Israel

[2] Department of Chemistry and Institute for Nanotechnology and Advanced Materials, Bar-Ilan University, Ramat-Gan, Israel

[3] Department of Chemistry, Ben Gurion University, Beer-Sheva, Israel

[4] Departments of Chemical Research Support, Weizmann Institute of Science, Rehovot, Israel

[*] doron.naveh@biu.ac.il

Process Protocol

1. Both growth procedures (fast and slow) start with N_2 blow (160 sccm) at 200 °C for drying the quartz tube and sample for 60 minutes and thereafter sulfur was heated to 150 °C. The temperature remained at 200 °C and N_2 flow was adjusted to 100 sccm for a period of 30 minutes, while the sulfur liquefies.

2. The fast growth on thin film Mo on Si/SiO_2 was performed at a temperature of 800 °C for 2 hours, with a steady temperature ramp of 20 °C/min and a flow of 30 sccm. After two hours the temperature is set to 600 °C for 30 minutes and the sulfur solidifies. The sample is then cooled to room temperature while washing the sulfur from the tube at a flow of 30 sccm.

3. The slow growth on thin film Mo on Si/SiO_2 was performed at a temperature of 800 °C for 2 hours, with a steady temperature ramp of 5 °C/min and a flow of 30 sccm. After two hours the temperature is set to 600 °C for 30 minutes and the sulfur solidifies. The sample is then cooled to room temperature while washing the sulfur from the tube at a flow of 30 sccm.

4. The growth on Mo foil started similarly with procedure #1 and then the temperature is set to 500 °C in a ramping rate of 20 °C/min, at which the sample is kept for 30 minutes. The sample is then heated to 600 °C at rate of 20 °C/min and remains in 600 °C for additional 30 min. Finally, the temperature is set to 750°C in a heating rate of 20 °C/min and remains at this temperature for 24 hours at a flow of 30 sccm. After the growth, the sample is cooled to 600 °C and remains in this temperature for 30 min while the sulfur solidifies and then it is cooled to room temperature while washing sulfur with N_2 flow of 30 sccm.

The schematic representation of the growth process of the slow and fast ramping samples grown on thin film Mo on Si/SiO_2 is provided in fig. S1 below. Fig. S2 shows corresponding conditions for the growth on thick Mo foil, fig. S3 displays a TEM cross section images of the sampe grown on thick Mo foil.

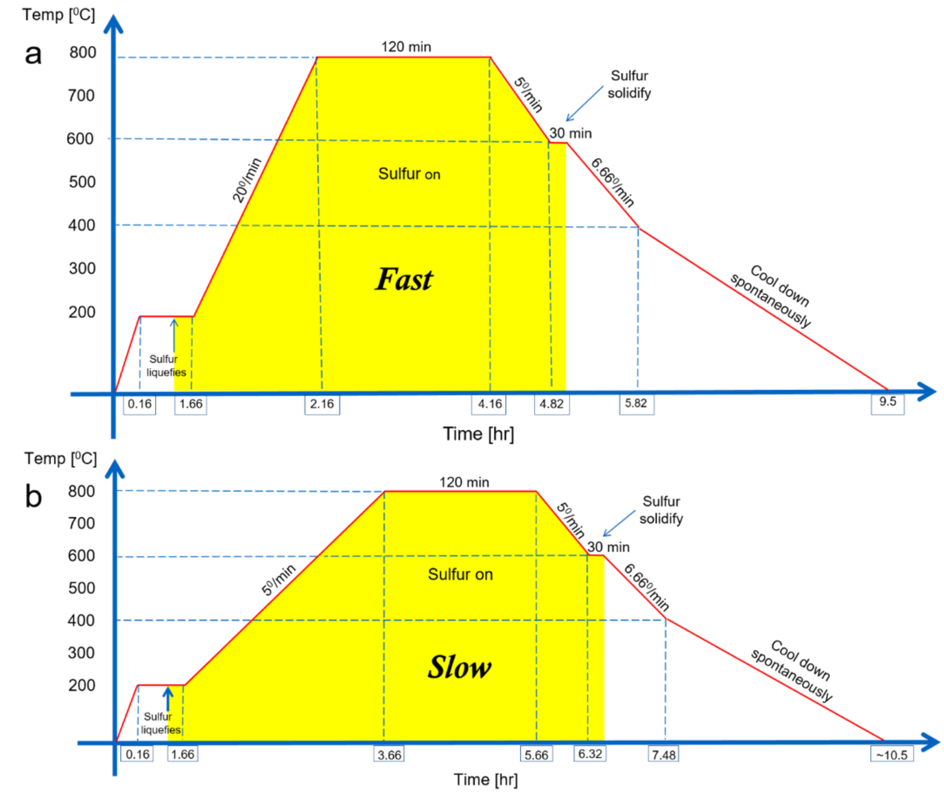

Figure S1. Process protocol of the growth on of MoS_2 by sulfurization of Mo thin film on a Si substrate with fast (a) and slow (b) temperature ramping.

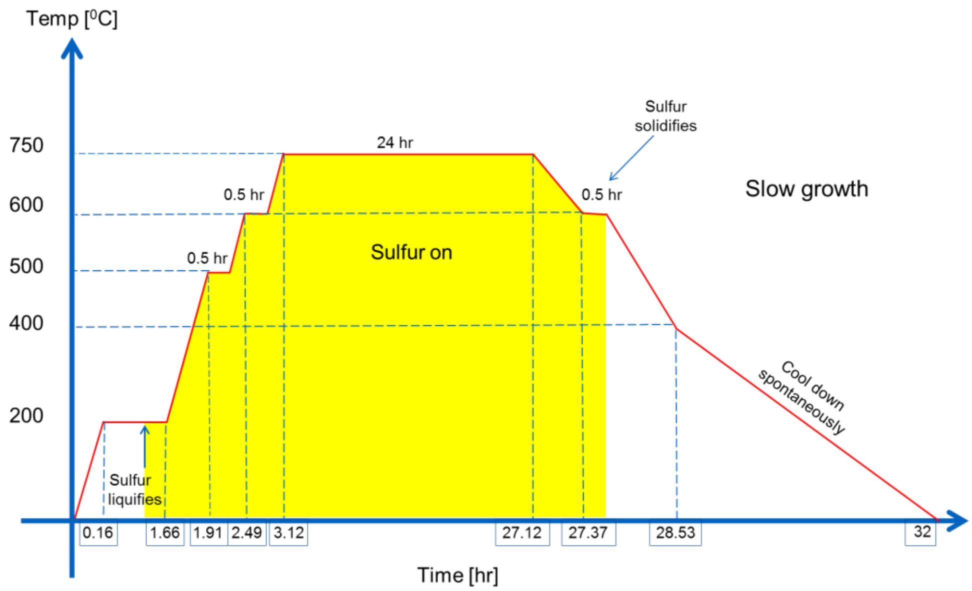

Figure S2. Process protocol of the growth of MoS_2 by sulfurization of a Mo foil.

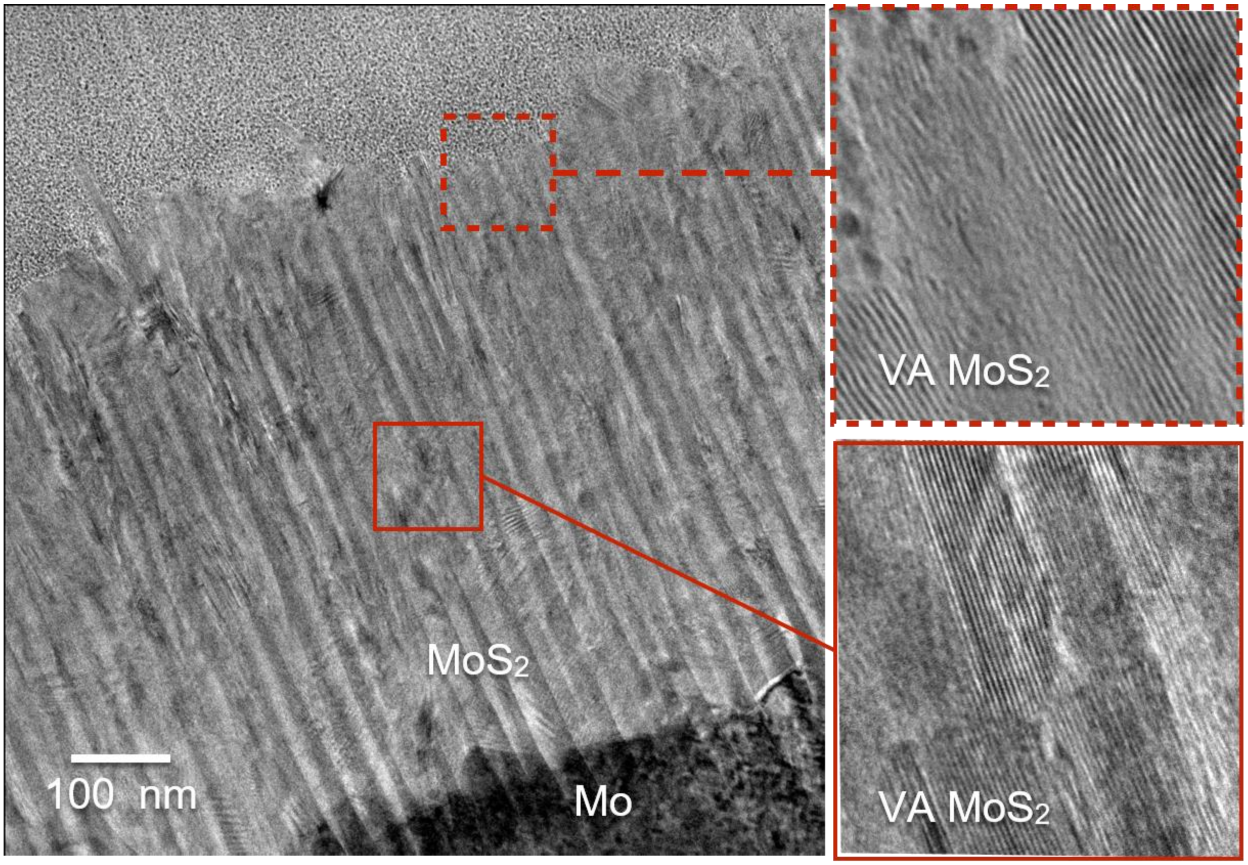

Figure S3: Slow growth condition with low temperature ramp on Mo (750 °C, 24h). On thick (25 µm) Mo foils the long reaction (24 h) results in a MoS_2 film thickness that is limited to the range of 1µm.

Analysis of Orientation Order

Fig. S4 outlines the procedure for the quantification of the orientation distribution of MoS_2 domains as a function of the film depth below the top surface. The procedure is built upon mapping the lattice reflections of basal planes of MoS_2 corresponding with lattice fringes in in a polar plot.

With the procedure a comparison of the crystal orientation parallel to the growth direction for two samples, grown at fast and slow temperature ramping rates, was done. Results are shown in Fig. S5. Fig. S5(a) displays orientation histograms for the fast growth, and fig. S5(b) for the slow growth. Fig. S5(c) displays the comparison of the orientation ordering as a function of the depth below the top surface for both conditions. Under slow growth conditions the top surface layer with randomly oriented crystal is substantially thinner than at fast growth conditions.

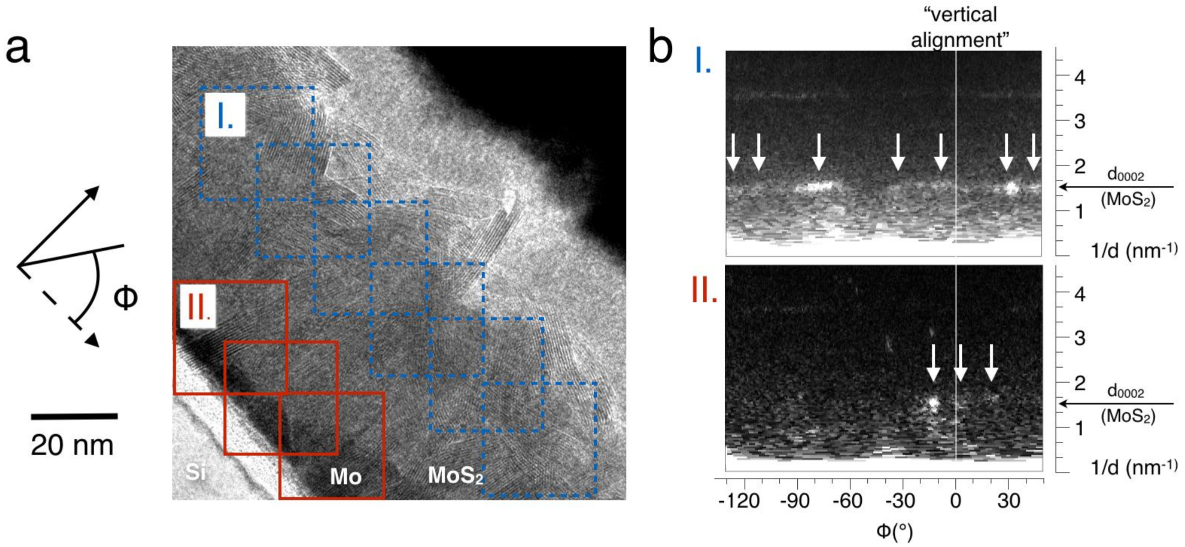

Figure S4. Quantification of orientation order for VA-MoS_2 grown on Si substrate at 800 °C, 2h (fast growth). (a) Lattice plane orientations in subframes of HRTEM images show up as spots in the (b) FFT of the subframes. Plotting the FFT in polar coordinates conveniently gives access to the rotation angle Φ of a crystal relative to the substrate plane. In (b) the FFT of the subframes grouping to regions I and II in (a) were added. Counting crystal orientations into bins of a histogram provides the orientational distribution of MoS2 basal planes.

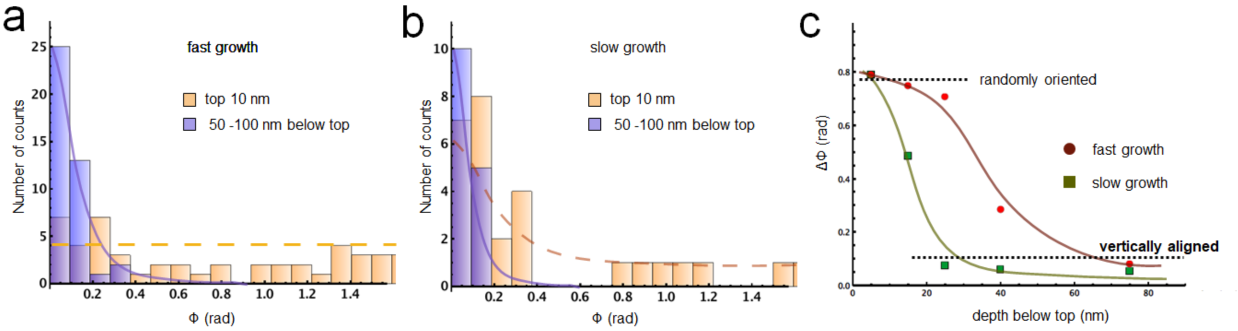

Figure S5. Statistical analysis of orientational ordering for VA-MoS_2 grown on Si substrate. Histogram plot of MoS_2 crystal orientations measured from the alignment of basal planes from HRTEM images. Data is shown for the depth ranges within the top 10 nm and between 50 nm to 100 nm for (a) fast growth and (b) slow growth conditions. At a later stage of the growth and larger depth (blue) a strong preferred orientation around the vertical alignment (Φ=0) is observed for both conditions. For the early growth (orange), up to 10 nm below the top surface, the fast growth conditions are characterised by a random orientation. Contrary, at slow growth conditions the orientation distribution peaks around the vertical alignment at Φ=0 even for the early stages of growth. (c) Angular spread of crystal orientations around the vertical alignment. ΔΦ is the angular spread around around the direction of vertical alignment at Φ=0 that contains 68 % of all orientations for a given depth below the top surface. Under slow growth conditions (green) the top surface layer with randomly oriented crystal is substantially thinner than at fast growth conditions (red). Lines are a guide to the eye.

Model for Mo Sulfurization I – Surface Reaction of the Disoriented Film

A moving boundary diffusion problem can well describe the sulfurization process of MoS_2, where sulfur vapor is the source for a reaction starting at the surface of a metal film. The sulfur concentration at the sample surface, C_0 depends on the partial pressure of sulfur vapor of sulfur in the reaction chamber. At the early stage of the reaction, sulfur penetrating the Mo surface and is assumed to be completely consumed, forming MoS_2 at an advancing reaction path. Here $C_{RMoS_2}(x,t)$ and $C_{Mo}(x,t)$ are the sulfur concentrations in the randomly oriented MoS_2 surface reacted layer (at $0 \le x \le x_0[t]$) and in Mo ($x > x_0$), respectively. Correspondingly, the diffusion coefficient of sulfur at the two media are D_{RMoS_2}, D_{Mo} and the model of diffusion-reaction process takes the form[1]:

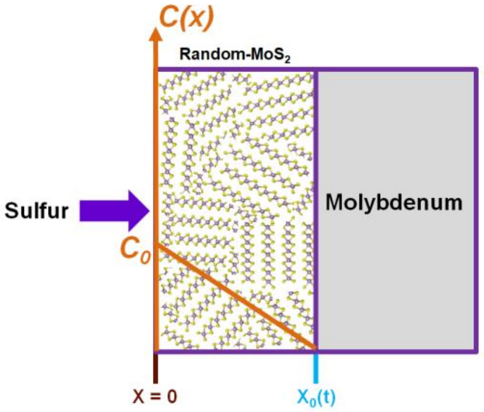

Figure S6. Schematic representation of growth front propagation. The sulfur concentration profile (indicated by orange line) drops from the surface to the interface with Mo.

$$D_{RMoS_2}\frac{\partial^2 C_{RMoS_2}}{\partial x^2} = \frac{\partial C_{RMoS_2}}{\partial t}, \qquad 0 \le x \le x_0(t) \qquad S1$$

$$D'_{Mo}\frac{\partial^2 C_{Mo}}{\partial x^2} = \frac{\partial C_{Mo}}{\partial t} + \frac{\partial \eta}{\partial t} = (1+R)\frac{\partial C_{Mo}}{\partial t}, \qquad x > x_0(t)$$

Here we assume equilibrium growth such that Mo consumption at every instant is proportional to the concentration of the diffusing sulfur by $\eta_{Mo} = RC_{Mo}$ and by definition $D_{Mo}\frac{\partial^2 C_{Mo}}{\partial x^2} = \frac{\partial C_{Mo}}{\partial t}$ and the effective diffusion constant of sulfur in Mo is $D_{Mo} = \frac{D'_{Mo}}{1+R}$. We assume initial conditions of $x_0(0) = 0, C_{RMoS_2}(x,0) = C_{Mo}(x,0) = 0$. The following boundary conditions were applied to solve this problem, assuming a steady-state growth (*i.e.*, equal flux of sulfur from the two sides of the boundary):

$$D_{RMoS_2}\frac{\partial C_{RMoS_2}}{\partial x} + C_{RMoS_2}\frac{dx_0}{dt} = D_{Mo}\frac{\partial C_{Mo}}{\partial x} + C_{Mo}\frac{dx_0}{dt}, \quad at\ x = x_0(t), \tau > t > 0 \qquad S2$$

The sulfur mass preservation condition is associated here with the reaction rate and with the total density of consumed sulfur atoms in the surface reaction, *N* (that can be approximated with the average density of MoS_2 to ~ 3.76×10^{22} cm^{-3})

$$N\frac{dx_0}{dt} = D_{RMoS_2}\frac{\partial C_{RMoS_2}}{\partial x}\ , \quad x = x_0(t)\ , \tau > t > 0 \qquad S3$$

A fixed boundary value is assumed at the sulfur source side, $C_{RMoS_2}(0,t) = C_0$, and also a full consumption of sulfur: $C_{Mo}(x > x_0, t) = 0,\ t < \tau$, where τ is the bounded surface-reaction time, that is self-limited.

The solution for the diffusion problem is given by Eq. S4a and resulting from it is the reaction rate defined by the velocity of the advancing reaction front as in Eq. S4b:

$$C_{RMoS_2}(x,t) = C_0\left[1 - \frac{\mathrm{erf}\left(\frac{x}{2\sqrt{D_{RMoS_2}t}}\right)}{\mathrm{erf}\left(\frac{x_0(t)}{\sqrt{D_{RMoS_2}t}}\right)}\right] \qquad S4a$$

The assignment of Eq. S4a into the rate equation S3 yields the exact solution that is plotted in Figure 3b.

$$\frac{dx_0}{dt} = \frac{C_0}{N}\sqrt{\frac{D_{RMoS_2}}{\pi t}}\ \frac{\exp\left(-\frac{x_0^2}{4D_{RMoS_2}t}\right)}{\mathrm{erf}\left(\frac{x_0}{2\sqrt{D_{RMoS_2}t}}\right)} \qquad S4b$$

Model for Mo Sulfurization II – Diffusion-limited Growth of VA-MoS_2

After some time τ from the beginning of the reaction, where the growth had generated randomly oriented crystals, enough crystal seeds are oriented along the crystal c-axis. These crystals can deliver sulfur to the reaction front along their van-der Waals gap that is aligned with the concentration gradient direction. Since diffusion along the c-axis is much faster, the kinetics of the reaction front aligns with this orientation so the sample is devised to three domains of randomly oriented MoS_2, vertically oriented MoS_2 and metallic Mo with corresponding diffusion coefficients and sulfur concentration C_λ, D_λ, λ = $RMoS_2$, $VMoS_2$, Mo, respectively, for each domain. For simplicity, we shift the coordinates $t' = t - \tau$, $x' = x - x_0$ from this point and the new reaction front $x_1(t)$ starts from the shifted origin. Neglecting transient effects, we define the *time-dependent value* of boundary concentration at the interface of the disoriented/oriented films

$C_B \equiv C_{RMoS_2}(x_0, t > \tau) = C_{VMoS_2}(x_0, t > \tau) \approx C_0 erfc\left(\frac{x_0}{2\sqrt{D_{RMoS_2}t'}}\right)$ and assume that $D_{VMoS_2} > D_{RMoS_2}$. Here again we have the same type of boundary conditions satisfying steady state growth:

$$\mathrm{C}_{VMoS_2}\frac{dx_1(t')}{dt'}+\mathrm{D}_{VMoS_2}\frac{\partial \mathrm{C}_{VMoS_2}}{\partial x'}=\ \mathrm{D}_{Mo}\frac{\partial C_{Mo}}{\partial x'}+\mathrm{C}_{Mo}\frac{dx_1(t')}{dt'} \qquad \mathrm{S5}$$

And initial conditions $\mathrm{C}_{VMoS_2}(t'=0)=\ \mathrm{C}_{Mo}(t'=0)=0$. The solution for the diffusion with the boundary conditions of complete reaction (i.e. no unreacted sulfur inclusions exist in Mo):

$$C_{VMoS_2}(x,t)=C_0 erfc\left(\frac{x_0}{2\sqrt{D_{RMoS_2}t'}}\right)\left(1-\frac{\mathrm{erf}\left(\frac{x-x_0}{2\sqrt{D_{VMoS_2}(t')}}\right)}{\mathrm{erf}\left(\frac{x_1(t')-x_0}{2\sqrt{D_{VMoS_2}(t')}}\right)}\right), x_1(t')>x>x_0\,, t'>0 \qquad S6a$$

and the reaction rate of the vertically aligned crystal is found by applying Eq. S3 to the expression of Eq. S6a:

$$\frac{dx_1}{dt}=\sqrt{\frac{D_{VMoS_2}}{\pi(t')}}\frac{C_0}{N}erfc\left(\frac{x_0}{2\sqrt{D_{RMoS_2}t'}}\right)\frac{e^{-\left(\frac{x_1-x_0}{2\sqrt{D_{VMoS_2}(t')}}\right)^2}}{\mathrm{erf}\left(\frac{x_1-x_0}{2\sqrt{D_{VMoS_2}(t')}}\right)} \qquad S6b$$

The solution of Eq. S6b as shown in figure 3c in the main document where the value of the diffusion constant ratio $\mathrm{D}_{VMoS_2}/\ \mathrm{D}_{RMoS_2}$ was assumed to be 200 and a rough estimation of $\mathrm{D}_{RMoS_2}\approx 10^{-13}\ cm^2/s$ was applied for constructing Figure 3c. [2,3]

Notably, at the limit of long diffusion times on thick enough samples, that are free from boundary constraints, the reaction is self-limited. This corresponds to the boundary conditions of a semi-infinite sample where the surface of the sample is kept at a fixed sulfur concentration, ($\mathrm{C}[0,\mathrm{t}]=\ \mathrm{C}_0$). The thickness of the grown MoS_2 layer defines an upper bound to the gradient of the sulfur concentration. Since the driving force for the diffusion is the gradient in sulfur concentration, the reaction rate decelerates with time, as the MoS_2 film grows thicker. This process is supported by the model, provided by Eq. S6b, whereat large x_1 values the reaction rate decays very fast. Experimentally, we found that on thick (25 µm) Mo foils the long reactions (24 h and beyond) results in MoS_2 film thickness that is limited to the range of 1µm, as in Fig. S3.

During growth with a linear temperature ramping the diffusion coefficient increases exponentially (see ref S1) and higher diffusivity of sulfur leads to a more rapid reaction at the Mo interface with sulfur vapor. This surface reaction can be assumed to start at some critical temperature, where the diffusion coefficient of sulfur becomes large enough to drive a reaction at the interface of solid Mo to produce randomly oriented MoS_2. The depth of this interface layer depends strongly on the temperature of the reaction that determines the sulfur penetration depth in the surface reaction. In this study, the temperature of the reaction is set to 800 °C - that is well above the temperature at which surface reaction is initiated.[16] Therefore, the early stages of the reaction at the Mo surface depend on the temperature ramping rate rather than the reaction temperature itself – that is reached at a point in time where surface reaction has been completed.

An approximate measure to the surface reaction time (at which reaction depth is ~Δx, the thickness of the random layer) can be approximated to $\Delta t \approx \left[\frac{\Delta x}{\sqrt{D}}\right]^2$ $(and\ \Delta x \approx$ 10nm, $D \approx 10^{-14} cm^2/s)$ - that is in the order of one minute. Therefore the *average temperature* during the formation of the random layer can be approximated with the initial and final temperatures at which the layer has reached a thickness of Δx - that is dominated by the temperature ramping rate.

The dominant parameter for controlling the thickness of the random layer, Δx, is the diffusion coefficient (here we fixed the pressure). By driving the reaction at a temperature (800 °C) well above the critical temperature at which a reaction can be initiated, the knob controlling the surface reaction is the temperature ramping rate and the effective diffusion rate that is associated with it, D_{Mo}.

References

1 Crank, J. *The mathematics of diffusion*. (Oxford university press, 1979).

2 Mrowec, S. & Przybylski, K. Defect and transport properties of sulfides and sulfidation of metals. *High Temperature Materials and Processes* **6**, 1-80 (1984).

3 Lee, B. S. & Rapp, R. A. Gaseous Sulfidation of Pure Molybdenum at 700°–950° C. *Journal of the Electrochemical Society* **131**, 2998-3006 (1984).
